# A novel 11p13 microdeletion encompassing *PAX6* in a Chinese Han family with aniridia, ptosis and mental retardation

**DOI:** 10.1186/s13039-015-0110-2

**Published:** 2015-01-22

**Authors:** Ping Hu, Lulu Meng, Dingyuan Ma, Fengchang Qiao, Yan Wang, Jing Zhou, Long Yi, Zhengfeng Xu

**Affiliations:** State key Laboratory of Reproductive Medicine, Department of Prenatal Diagnosis, Nanjing Maternity and Child Health Care Hospital Affiliated to Nanjing Medical University, 123# Tianfei Street, Baixia District Nanjing, 210029 China; Department of Pathology, Nanjing University Medical School, Nanjing, China

**Keywords:** *PAX6*, 11p13 deletion, Mental retardation, Aniridia, SNP-array

## Abstract

**Purpose:**

To explore possible genetic aberrations in a Chinese family with aniridia, ptosis and mental retardation, and provide genetic evidence for the prenatal diagnosis.

**Methods:**

14 exons of PAX6 in the proband were sequenced by the Sanger sequencing technique. Multiplex ligation-dependent probe amplification (MLPA) technique was employed to further explore gene alterations of *PAX6*. Single nucleotide polymorphisms-array (SNP-array) assay was applied to screen potential pathologic genome-wide copy number variations (CNV).

**Results:**

There were no detectable pathogenic mutations in the 14 exons of PAX6 in the proband. MLPA indicated a heterozygous deletion encompassing all PAX6 gene regions covered and a partial upstream region. SNP-array assay detected a heterozygous 11p13 microdeletion with a length of 518 kb in the proband, spanning two whole annotated genes, elongation factor protein 4 (*ELP4*), the paired box gene 6 (*PAX6*), and partial IMP1 inner-mitochondrial membrane (*IMMP1L*) gene. SNP-array revealed her affected brother carried the identical deletion.

**Conclusions:**

The 518 kb heterozygous deletion in 11p13 encompassing *PAX6* should be the genetic etiology for the familial aniridia.

## Background

Congenital aniridia (OMIM 106210) is a kind of eye disorder characterized by complete or partial hypoplasia of the iris. The worldwide prevalence of aniridia was estimated to be 1:50 000 to 1:100 000 in the year of 2013 [1]. About two-thirds of reported aniridia cases were familial and showed a dominant inheritance manner with nearly complete penetrance, the remaining one-third were sporadic [2]. *PAX6* (OMIM 607108), a well-known aniridia disease-causing gene located in chromosome 11p13 region, contains 14 exons and encodes a protein of 422 amino acids. As a transcriptional factor, *PAX6* is involved in the development of a diversity of tissues and organs including ocular tissues, olfactory bulb, neural tube, gut and pancreas [3,4]. Heterozygous loss of function of PAX6 was identified in about 90% aniridia cases, with intragenic mutations accounting for approximately two-thirds of cases and chromosomal rearrangements accounting for the other one-third [2].

Previous studies demonstrated that people harboring *PAX6* mutation displayed a clinical symptom spectrum including aniridia, corneal opacification, keratitis, cataract, glaucoma, lens dislocation, ciliary body hypoplasia, foveal hypoplasia, strabismus, nystagmus, Peter’s anomaly, optic nerve defects [5], hyposmia, abnormal inter-hemispheric auditory transfer [6], impaired islet function [7] and brain structure abnormalities [8]. Until now, approximately 357 intragenic mutations have been documented in the *PAX6* mutation database (http://www.hgu.mrc.ac.uk/Softdata/PAX6/), while microdeletion cases associated with *PAX6* region were rare.

In this study, we report on a novel genomic microdeletion including *PAX6* in a small Chinese family with aniridia, cataract, ptosis and mental retardation detected by SNP-array assay.

## Case presentation

**Figure 1:**
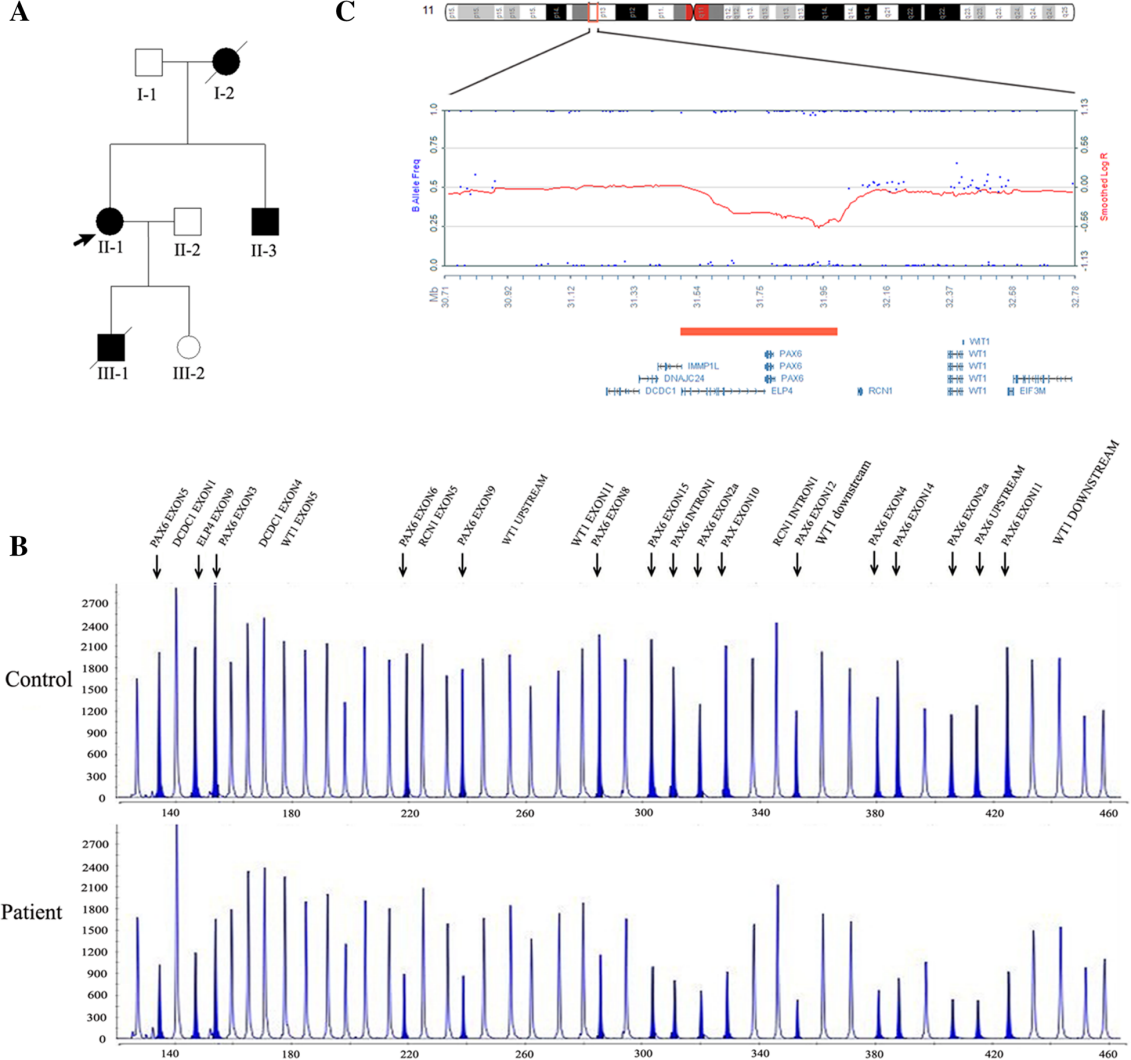
**11p13 microdeletion in the family with aniridia.** **A**. Pedigree of the family. Squares and circles indicated males and females respectively. The symbols in black represent the affected members. The arrow indicates the proband. The square with a line indicated a deceased individual. The inheritance pattern in the family is autosomal dominant. **B**. MLPA results from a normal control and the proband (II:1). These peaks filled with solid blue represent probes that display nearly half dose reduced. **C**. The 518 kb genomic microdeletion of chromosome 11p13 identified by SNP-array. This deletion harbors two whole genes ELP4, PAX6, and the first two exons of IMMP1L.

This three-generation family included four affected patients, two of them were available for this study (Figure 1A).

The proband (II:1), a 31-years-old pregnant Chinese Han female who suffered from congenital bilateral complete absence of iris, cataracts, bilateral ptosis was referred to our center for genetic counseling at 7 weeks' gestation. She is the first child born to a non-consanguineous couple. Her father is healthy while her mother (I:1) and bother were suffered by anirida. She is normal in growth and speaking during her development. She did not display any intellectual disability. Her first child, who was also affected by anirida, was born when she was 24. Her mother and first child deceased at the age of 45 and 5 due to non-medically related causes. Serum growth hormone (GH), thyroid stimulating hormone (TSH), free triiodothyronine (fT3), and free thyroxineIndex (fT4) levels were all in normal range. Ultrasonography results indicated her internal organs were normal.

Her affected brother (II:2) of 26-years-old was also recruited for clinical tests. He is normal in height and weight. Physical examination was normal, while minor anomalies including prominent forehead, short philtrum were observed. He was diagnosed with mental retardation when he was 7 years-old. His IQ was 70 when he was 17 years-old. Ultrasonography results indicated his internal organs were normal. MRI did not reveal any structure abnormalty.

## Results

### Mutation analysis for PAX6

Mutation screening of 14 *PAX6* exons by Sanger sequencing did not reveal any abnormality in the proband (data not shown).

### Multiple ligation-dependent probe amplification (MLPA) assays

MLPA assays were used to identify possible *PAX6* microdeletion (Figure 1B). MLPA results revealed that the height of all these probes in the *ELP4* and *PAX6* displayed nearly half-dose reduced, whereas probes targeting genes up- or downstream to the deletion such as *DCDC1, RCN1,WT1*, exhibited nearly the same height as the normal. MLPA assay on amniotic fluid cells collected at the 18 gestation week revealed the fetus (III:2) did not inherit the deletion from the mother. The female baby was born at 39 weeks, and physical examinations confirmed no abnormalities especially in the eyes. Due to the unavailability of the DNA samples, we are unable to determine whether the deletion existed in patients (I:2, III:1).

### SNP-array assay

SNP-array test was applied to find possible pathologic copy number variations besides *PAX6* microdeletion in the proband and her brother. The results identified a heterozygous deletion in the 11p13 region expanding from 31,486,211 to 32,004,859 bp (hg18) with a length of 518Kb in the proband. The deleted genomic fragment contains two whole genes, elongation factor protein 4 (*ELP4*), the paired box gene 6 (*PAX6*), and partial IMP1 inner-mitochondrial membrane (*IMMP1L*) gene (exon1, 2). Additionally, an identical 11p13 deletion was identified in proband’s brother (Figure 1C). However, no other pathologic copy number variations were found in the genome-wide SNP-array scanning in both of them.

## Discussion

In this study, we characterized a novel 518 kb deletion on 11p13 in a three-generation Chinese Han family with familial aniridia. In addition to the aniridia and cataracts, interestingly, one of them also presented with mental retardation. We speculated that *PAX*6, which lies in the deleted region, primarily accounted for these clinical features.

Neurological disorders such as mental retardation [9], autism [10], epilepsy, cognitive impairments or behavioral abnormalities [11] are uncommon in individuals with *PAX6* mutations. Chromosome microdeletions were much less commonly reported in aniridia patients than *PAX6* point mutations. As far as we know, only 14 cases were found to carry genomic microdeletion encompassing *PAX6* or its regulatory region but not *WT1* to date [12-20], and only one of them displayed neurodevelopmental disorders. Davis LK et al. reported a 13-year-old patient with aniridia, autism, and mental retardation carrying a 1.3 Mb heterozygous microdeletion, approximately 35 kb distal to the last exon of *PAX6* [15]. Further study indicated the deletion was inherited from the mother who presented with aniridia, depression, anxiety, and social awkwardness. Though the copy number of *PAX6* was intact in the family, the regulatory genomic region of *PAX6* nearby *ELP4* included in the deletion was believed to be the etiology. In our study, a 518 kb submicroscopic deletion including *PAX6*, *ELP4* and partial *IMMP1L* was identified. All the four patients in the family were normal in other physical development, but the proband’s brother presented with moderate mental retardation, indicating a marked intra-familial variation.

Neurological disorders presented in individuals with PAX6 mutations can be explained by the *PAX6* expression pattern and outcome from animal model research. PAX6 expressed in the telencephalon, diencephalon, caudal rhombencephalon, myelencephalon and spinal cord, but not mesencephalon in the embryonic period, and adult brains [21-23], suggesting a role in the brain development. Pax6 mutant mice/rats displayed abnormal development in arealisation of the cerebral cortex, formation of the prosencephalon-mesencephalon boundary, axon guidance, differentiation of neurons from glia and neuronal migration in the cerebellum [24]. Brain defects were occasionally found to be related with PAX6 mutations. Magnetic resonance imaging (MRI) examination on patients with PAX6 mutations revealed a spectrum of brain abnormalities including absence/hypoplasia of the anterior commissure, reduced olfaction [25], polymicrogyria, absence of pineal gland [8], reduction in the white matter in the corpus callosum and grey matter in the anterior cingulated cortex, cerebellum, medial temporal lobe [26]. However, the patients’ brains were normal detected by brain MRI test in our case. The detailed molecular mechanisms underlying the mental retardation and brain structure defects of *PAX6* mutation is yet unknown.

Congenital aniridia can also occur in the WAGR syndrome (Wilms’ tumor, aniridia, genitourinary abnormalities, and mental retardation), a syndrome due to a contiguous gene deletion encompassing both *PAX6* and *WT1*. Individuals, particularly children under 6 years old, with *WT1* deletion may predispose to Wilms’ tumor, a kidney tumor with childhood onset [13]. Defining the accurate breakpoints of the 11p13 deletion may contribute to the genetic counseling, disease prognosis and prevention [1].

Another interesting gene is *EPL4*, which encodes a component of the six subunit elongator complex, a histone acetyltransferase complex that associates directly with the RNA polymerase II (Pol II) holoenzyme involved in transcriptional elongation. DNaseI hypersensitivity mapping and reporter transgenic assays revealed the presence of several putative cis-regulatory elements, including ones driving expression in lens and retina [27]. These elements reside within introns of the adjacent, ubiquitously expressed ELP4 gene, but are nevertheless thought to be PAX6-specific long-range control elements [28]. Andrea C et al. demonstrated that three SNPs in *ELP4* affect the size of the optic nerve head [29]. Zhang X et al. revealed that a 3' deletion to the *PAX6* gene including the *ELP4* was identified in one three-generation family with total aniridia [19]. A part of IMMP1L gene was also deleted in our patients. IMMP1L encode a subunit of IMP (mitochondrial inner membrane peptidase) complex which plays a role in the generating mature, active proteins in the mitochondrial intermembrane space by proteolytically removing the mitochondrial targeting presequence of nuclear-encoded proteins.

In this study, we accurately identified the pathologic genetic aberration of the family using SNP-array. In fact, consistent with our prenatal genetic diagnosis assistance, they delivered a healthy baby. The couple was informed they have a 50% chance to birth a healthy baby in subsequent pregnancies.

## Conclusions

In summary, this study identified a novel deletion containing *PAX6* in a Chinese family, expanding the mutation spectrum of *PAX6* aberration. The 518 kb heterozygous deletion of chromosome 11p13 may be the cause of the familial aniridia, congenital ptosis and slight mental retardation in this family. The function of the deleted genes are needed to be further studied. This work emphasizes the necessity to screen for possible microdeletion related with *PAX6* in patients with aniridia particularly when result of conventional sequencing analysis for *PAX6* is negative.

## Methods

### DNA extraction and sequencing

Genomic DNA was extracted from the venous blood of the two adult patients and cultured amniotic fluid fibroblasts. The 14 exons of PAX6 gene were amplified by polymerase chain reaction (PCR) using primers described by Redeker, et al. [10]. Obtained PCR products were sequenced bi-directionally using the ABI BigDye Terminator Cycle Sequencing kit v3.1 (ABI Applied Biosystems, Foster City, CA), according to the standard protocol.

### SNP-array assay

Human cyto12 SNP-array (Illumina, USA) comprising around 300,000 SNPs was applied for whole genome scan on the two affected individuals (the proband II-1 and her brother II-3). SNP-array tests were performed according to the manufacturer's protocol (Illumina, USA), chromosome karyotype analysis was carried out by KaryoStudio V 1.3.11 (Illumina, USA) and GenomeStudio V2011.1 (Illumina, USA) respectively. We use hg18 genome coordinate in this study.

### Multiplex ligation-dependent probe amplification (MLPA) technique

SALSA MLPA Kit P219-B2 PAX6 (MRC-Holland Amsterdam, Netherlands) was employed to verify the abnormal SNP-array findings and to test the DNA of the present fetus. The kit contains probes targeting each exon of PAX6 with the exception of exon 7 and 13, and multiple other genes within 11p13 region including BDNF, FSHB, DCDC1, ELP4, RCN1, WT1, LOC645981, LOC646008, HIPK3, LMO2, EHF, CD44 and SOX2. In brief, 100 ng DNA samples were denatured for 5 min at 98°C and then cooled to 25°C. Probes were mixed and hybridized with DNA samples at 60°C overnight and were then reacted with ligase 65 at 54°C for 15 min, followed by 5 min at 98°C and kept at 4°C. Finally, all probes and sample ligations were amplified by PCR using specific carboxyfluorescein (FAM) labeled PCR primers. Electrophoresis was performed using an ABI PRISM 3130 (Applied Biosystems) and Data was analyzed by GeneMarker V4.0 software. A peak area ratio between 0.7 and 1.3 times was considered as normal one, below 0.7 represents deletions and above 1.3 represents duplications. Each result was confirmed by two independent tests.

## Consent

Written informed consent was obtained from the patient’s parents for publication of this paper. A copy of the written consent is available for review by the Editor-in-Chief of this journal. This research was approved by the Ethics Committee of Nanjing Maternity and Child Health Care Hospital.
